# Multiple stage tissue expansion for reconstruction of scalp nevocellular nevus in pediatric age group

**DOI:** 10.3205/iprs000134

**Published:** 2019-04-24

**Authors:** Hiba AlBurshaid, Yasir Ali Alshehri, Lamya AlAbdulrahman, Reem AlJehani, Hussein Fadaak

**Affiliations:** 1 Department of Plastic Surgery and Burn Unit, King Fahad University Hospital, AlKhobar, Saudi Arabia

## Abstract

**Aim:** To present a rare case of giant congenital nevocellular nevus in a 7-year-old girl’s scalp and to highlight our management steps and outcomes.

**Case description:** An otherwise healthy 7-year-old girl presented to plastic surgery clinic with a giant congenital nevus (GCN) that covered almost her entire scalp that was treated successfully with tissue expander three times over a period of 14 months. A total of 34 cm width of skin, which comprised 78% of the patient’s scalp, was removed. The patient was reassessed five years later with a great cosmetic outcome represented by a remarkable hair growth and near normal scalp appearance.

**Conclusion:** Giant scalp nevocellular nevi in pediatric age group can be treated completely with tissue expanders more than twice to achieve near normal outcomes.

## Introduction

**Figure 1:**
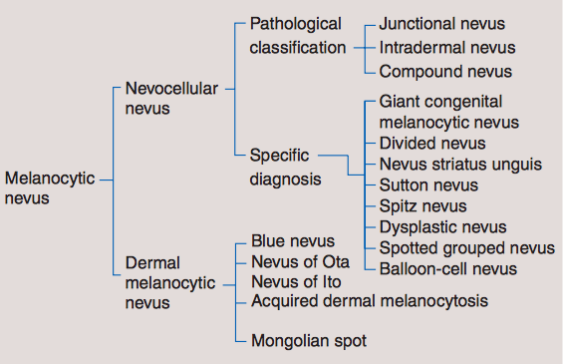
Different types of nevocellular nevus [1]

Nevocellular nevi are benign melanocytic tumors that originate in the skin. They are usually congenital and a result of proliferation of melanocyte in the skin. One type of congenital nevi is giant congenital nevus (GCN) (Figure 1 (Fig. 1)), which covers a large area of the body due to overpigmentation and causes a great cosmetic concern to the patient [1], [2]. In general, congenital nevi affect 1% of newborns in terms of small size nevi. However, this is not the case with GCN, the incidence of it is far less than the incidence of large congenital nevi, which occur to 1 in 200,000 babies [3]. GCN does not only cause a cosmetic concern, but also places the patient at a risk of 5–7% to develop melanoma. Therefore, early excision and reconstruction by plastic surgeons is necessary [1], [2].

For such cases, surgical treatment is performed to remove the lesion. It can range from resection, graft to usage of tissue expanders (TE) [4].

## Case description

 A 7-year-old girl with no significant past medical, surgical or family history was referred to our plastic surgery clinic as a case of a pigmented nevus that involved most of the scalp since birth. The nevus was associated with pruritus and serous discharge. 

**Figure 2:**
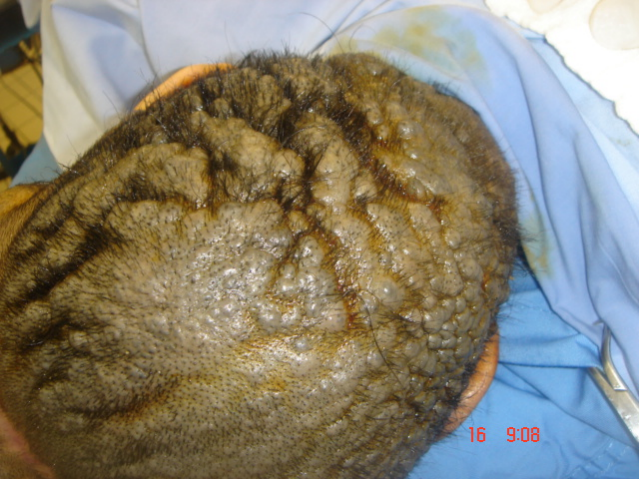
Picture shows the involvement of a pigmented and corrugated nevus on the scalp.

**Figure 3:**
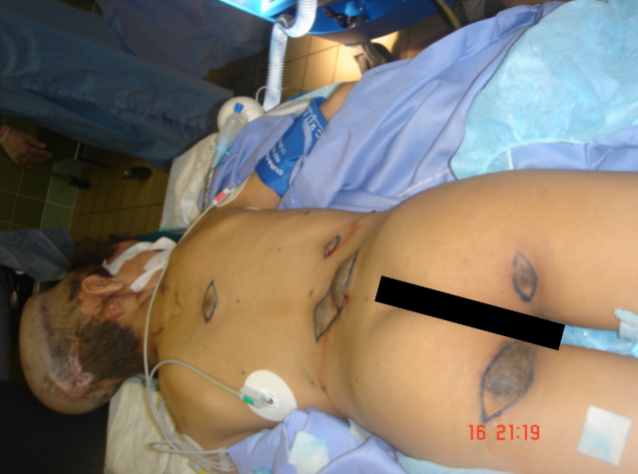
Hyperpigmented lesions, 4 on the back and 2 on the thighs with different sizes. All lesions were full of hair and weren’t secreting any discharge.

Local examination revealed a pigmented, thick, and corrugated lesion that involved almost her entire scalp leaving a strip of normal skin with a width of 10 cm (Figure 2 (Fig. 2)). On full body examination, she was found to have scattered, hyper-pigmented lesions fully covered with hair on the lower back and the upper thighs with estimated sizes ranging from 2x 2 to 6x 6 cm (Figure 3 (Fig. 3)). 

**Figure 4:**
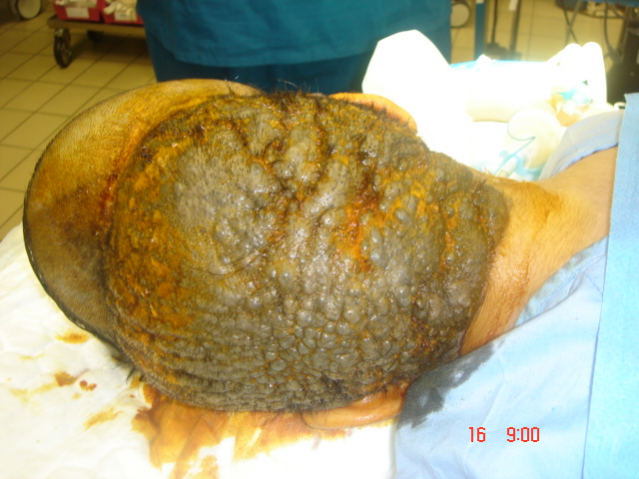
The first tissue expansion was inserted in the scalp.

**Figure 5:**
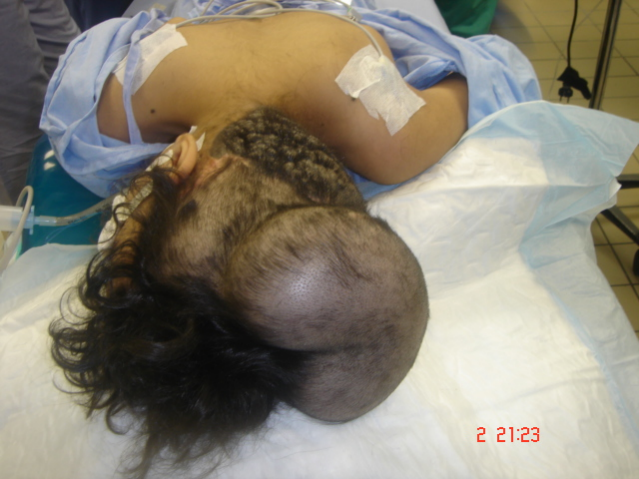
Insertion of second tissue expander

**Figure 6:**
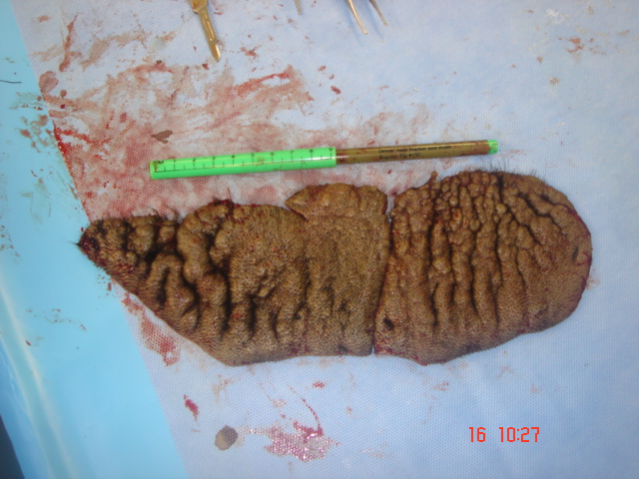
The excised skin of the patient’s scalp after the second procedure

**Figure 7:**
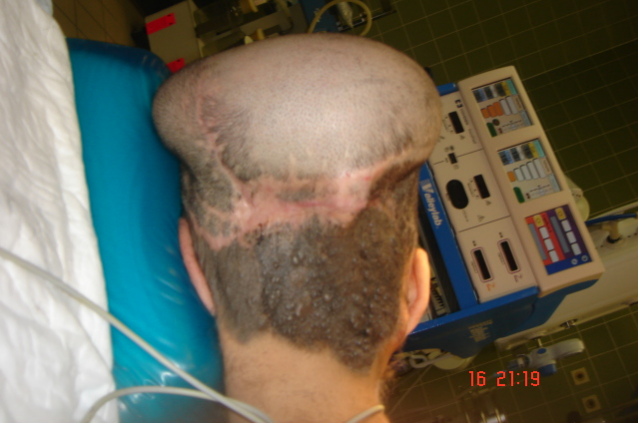
The third and last tissue expander placed in the scalp

Summary of the procedures done is shown in Table 1 (Tab. 1), Figure 4 (Fig. 4), Figure 5 (Fig. 5), Figure 6 (Fig. 6), and Figure 7 (Fig. 7) show single procedures.

**Figure 8:**
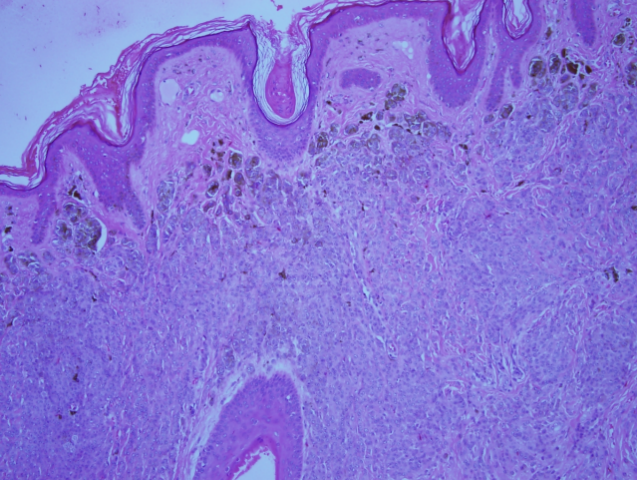
Higher power shows dermal nests with marked adnexocentricity, in addition to infiltrating between the collagen bundles.

**Figure 9:**
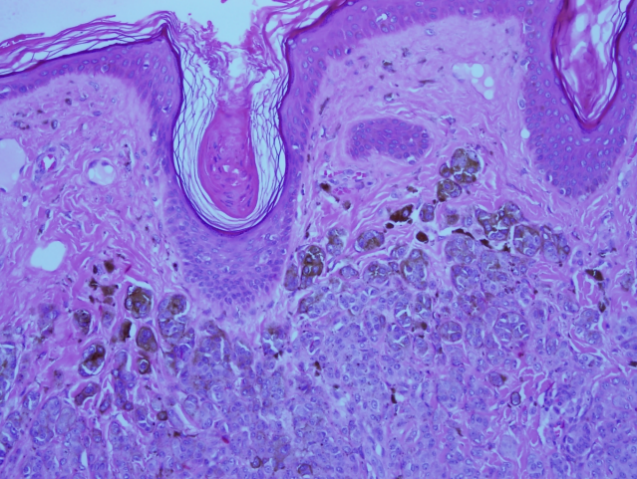
High-power view shows intact dermo-epidermal junction with pigmented melanocytic nests in the superficial part of the nevus.

All the specimens collected were sent to the histopathology lab and the report revealed that the specimens had nests and diffusely cellular sheets of benign melanocyte with superficial focal pigmentation and deep dermal maturation. Lateral margins and deeper margins were involved. Overall, there was no malignancy reported (Figure 8 (Fig. 8), Figure 9 (Fig. 9)). 

**Figure 10:**
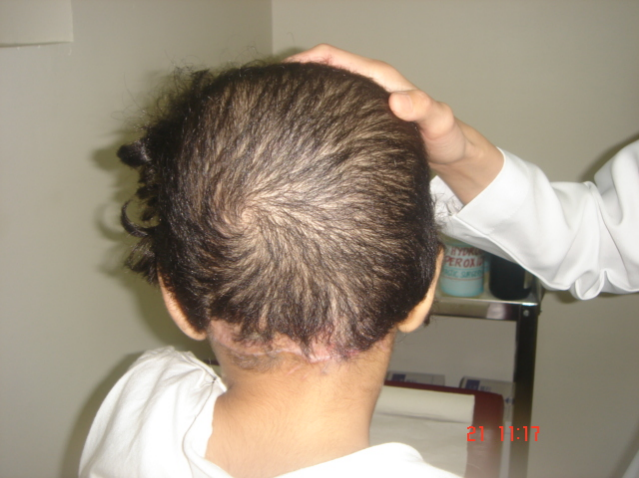
Picture shows good hair growth in the occipital region 3 months after the last procedure

Figure 10 (Fig. 10) shows the result 3 months after the last procedure. 

**Figure 11:**
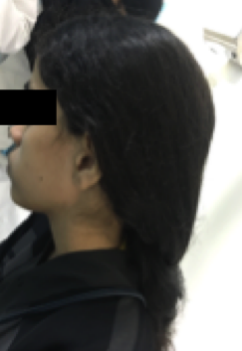
Five years later, the patient with a perfect hair growth that reaches mid back with no complications

Five years later and at the age of 13, she was seen with her parents in the clinic doing fine with a normally growing long hair and no complications (Figure 11 (Fig. 11)).

## Discussion

Tissue expander is a technique used for stretching the tissue to a certain amount for reconstruction. It involves using an expandable balloon filled with a fluid beneath the tissue to be expanded repeatedly [5], [6]. 

It’s been observed that the complication rates increase with repeated usage of TE on the same tissue multiple times [7]. Tissue expansion is considered to be the optimal method used to grow skin with the same characteristics as the surrounding normal skin in terms of color, texture, and thickness and to create as minimal scars as possible and reduce the risk of rejection [8]. Furthermore, for new skin to be created, a sequence of signaling pathway should be activated. This is only possible when the skin is put under tension to be stretched to a certain level with the help of TE [8]. In general, it is considered to be a safe procedure. However, caution must be taken when used in the pediatric age group due to possible negative effects on the craniofacial growth [6]. In our case, TE was used thrice with no complications noted on the patient. In addition, according to LoGiudice et al. [7], in patients with hair loss more than 50%, using TE is not efficacious. However, in our case, the patient’s hair loss was approximately 78%, yet TE was used successfully.

Also, it has been noted that excision of nevus is optimally done at childhood due to the great risk it imposes for the nevus to be transformed into a malignant one [9]. To clarify, 70% of the patients with a giant congenital melanocyte experience malignancy by the age of thirteen years. Also, those patients have a 51.4% greater risk to develop melanoma than the rest of the population [9]. Despite the fact that many options are available for excision of a nevus, surgical excision of a congenital nevus remains to be the most effective [10]. 

The disadvantages of multi-staged TE for treating this case include multiple hospital admissions and the complications that may arise from anesthesia and the surgery. Another disadvantage would be the long period it takes to see the final outcome. 

Nonetheless, this technique provides excellent functional and cosmetic outcome for lesions that occupy a large area of the body.

## Conclusions

Tissue expander is widely used to construct areas of the body where grafts cannot be used. In general, it is considered to be a safe procedure. However, when used in pediatric age group, care must be taken into consideration. Due to its effect on the growth of the cranium, tissue expanders are usually not used more than twice in children in most cases. In our case, TE was used thrice with no complications noted on the patient. Thus, using it more than two times in certain conditions with children may result in favorable outcomes rather than disastrous ones.

## Notes

### Competing interests

The authors declare that they have no competing interests.

### Informed consent

Informed consent was obtained from the patient’s father.

## Figures and Tables

**Table 1:**
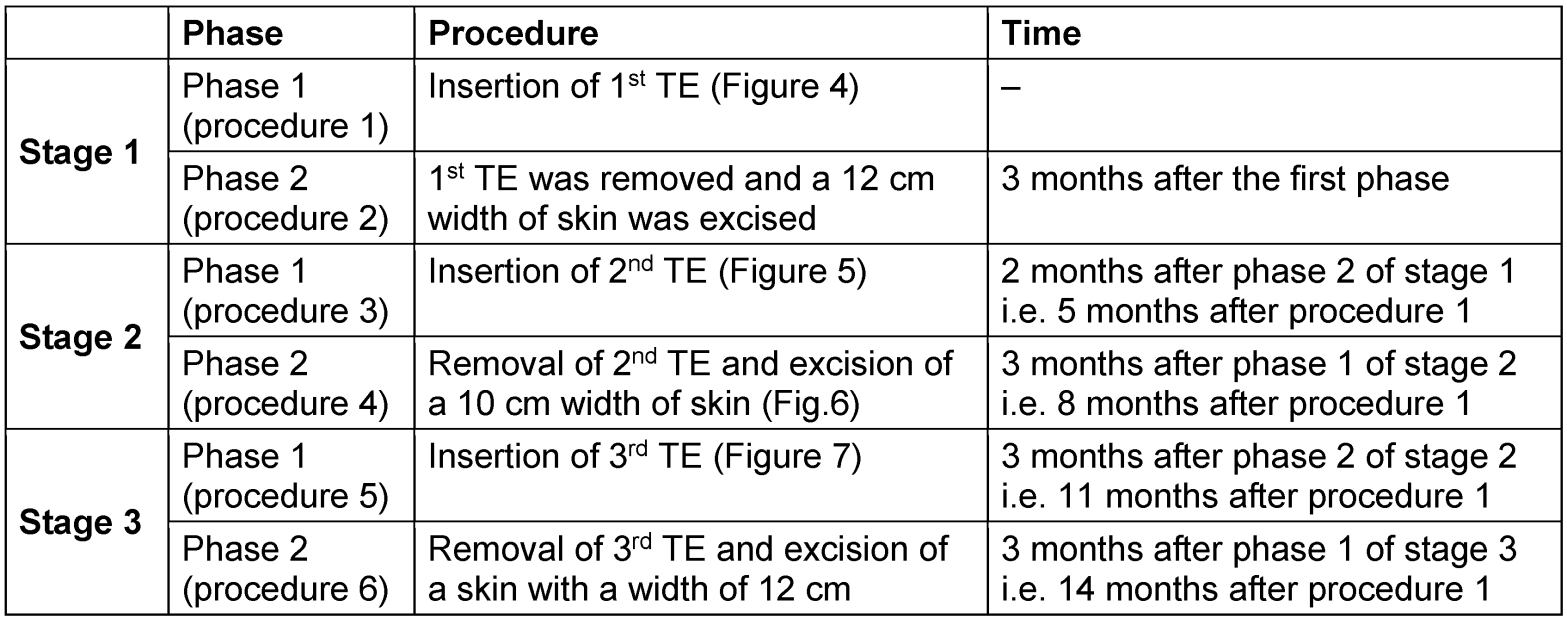
A time table that summarizes the main 3 stages of the surgery and each phase with a brief description of the procedures done along with their time with reference to the previous procedure
